# Tackling health inequalities: moving theory to action

**DOI:** 10.1186/1475-9276-6-12

**Published:** 2007-10-03

**Authors:** Louise Signal, Jennifer Martin, Papaarangi Reid, Christopher Carroll, Philippa Howden-Chapman, Vera Keefe Ormsby, Ruth Richards, Bridget Robson, Teresa Wall

**Affiliations:** 1 Department of Public Health, University of Otago, Wellington, New Zealand; 2 Te Rōpū Rangahau Hauora a Eru Pōmare, University of Otago, Wellington, New Zealand; 3 Ministry of Health, Wellington, New Zealand

## Abstract

**Background:**

This paper reports on health inequalities awareness-raising workshops conducted with senior New Zealand health sector staff as part of the Government's goal of reducing inequalities in health, education, employment and housing.

**Methods:**

The workshops were based on a multi-method needs assessment with senior staff in key health institutions. The workshops aimed to increase the knowledge and skills of health sector staff to act on, and advocate for, eliminating inequalities in health. They were practical, evidence-based, and action oriented and took a social approach to the causes of inequalities in health. The workshops used ethnicity as a case study and explored racism as a driver of inequalities. They focused on the role of institutionalized racism, or racism that is built into health sector institutions. Institutional theory provided a framework for participants to analyse how their institutions create and maintain inequalities and how they can act to change this.

**Results:**

Participants identified a range of institutional mechanisms that promote inequalities and a range of ways to address them including: undertaking further training, using Māori (the indigenous people) models of health in policy-making, increasing Māori participation and partnership in decision making, strengthening sector relationships with iwi (tribes), funding and supporting services provided 'by Māori for Māori', ensuring a strategic approach to intersectoral work, encouraging stronger community involvement in the work of the institution, requiring all evaluations to assess impact on inequalities, and requiring the sector to report on progress in addressing health inequalities. The workshops were rated highly by participants, who indicated increased commitment to tackle inequalities as a result of the training.

**Discussion:**

Government and sector leadership were critical to the success of the workshops and subsequent changes in policy and practice. The use of locally adapted equity tools, requiring participants to develop action plans, and using a case study to focus discussion were important to the success for the training. Using institutional theory was helpful in analysing how drivers of inequalities, such as racism, are built into health institutions. This New Zealand experience provides a model that may be applicable in other jurisdictions.

## Background

Tackling health inequalities has been recognised as a key goal for of the New Zealand government [1] and governments internationally [2-4]. In New Zealand, inequalities in health, and in the determinants of health, are pronounced and have been shown to be increasing, at least up until the last available figures in 1999[5,6]. They include inequalities between ethnic groups, people of different socio-economic status, geographic inequalities, inequalities of gender and inequalities experienced by people with disabilities[7,8]. Of particular concern is the nine and a half year life expectancy gap between Māori (the indigenous people) and non-Māori[6]. The New Zealand Ministry of Health (MoH) commissioned awareness-raising workshops for senior New Zealand health sector staff as part of its Government's overall goal of reducing inequalities in health, education, employment and housing. This paper reports on this initiative.

## Methods

The workshops were developed in partnership between academics of the Department of Public Health at the University of Otago, Wellington (UOW) and the MoH Reducing Inequalities Policy Team. They were based on a needs assessment with senior staff in the MoH and District Health Boards (DHBs) (key funders and providers of hospital and health services) that used documentary analysis, key informant interviews and focus groups to gather data[9]. The needs assessment found that the inequalities goal was highly visible in key sector documents, such as DHB strategic plans. Most staff acknowledged its importance, with a number arguing that the inequalities goal needs to be internalised into all work undertaken. Strong support for training in health inequalities was identified although some staff expressed a level of cynicism about whether 'anything can be done' to address inequalities, and a sense of being overwhelmed by the size of the challenge.

The purpose of the workshops, *Tackling Inequalities: moving theory to action*, was 'to increase the knowledge and skills of DHB and MoH staff to act on, and advocate for, eliminating inequalities in health in Aotearoa/New Zealand'. They were practical, evidence-based, and action oriented, as recommended in the needs assessment. Eight two-day workshops were held around the country: one for DHB Māori managers, two for MoH staff and five for staff and board members of groups of DHBs.

Consistent with international and national literature, the workshops took a social approach to the causes of inequalities in health[10,11], focusing on the unequal distribution of the determinants of health[12]. Critical drivers of this unequal distribution appear to be socioeconomic position and social exclusion, including racism [13-16]. The training used ethnicity as a case study to investigate inequalities, as it is a critical area in New Zealand[6]. Because of this, racism was used to explore issues of social exclusion. There are many theoretical explanations of the impact of racism on health, which Harris and colleagues summarise as including 'differential exposure to determinants of health – eg, socioeconomic, environmental, and behavioural – differential access to, and quality of, health-care services, and direct effects of racism, such as trauma and stress[16].

Jones' framework for understanding racism 'is useful for raising new hypotheses about the basis of race-associated differences in health outcomes, as well as for designing effective interventions to eliminate those differences'[17]. She argues that racism occurs at three levels – institutionalized, personally mediated and internalised. Institutionalized racism is defined as:

... differential access to the goods, services, and opportunities of society by race. ... it is structural, having been codified in our institutions of custom, practice, and law, so there need not be an identifiable perpetrator. Indeed, institutionalized racism is often evident as inaction in the face of need[17].

In order to explore how health institutions create and maintain inequalities in health we drew on institutional theory[18,19]. Proponents of this theory from political science and economics argue that 'institutions matter'; that institutions structure the development and implementation of policy, and therefore the programmes and services that result. The approach focuses on the dominant ideas built into institutions, their institutional structures, and the processes and rules by which institutions operate. In the workshops, institutional theory provided a framework for participants to analyse how the MoH and DHBs contribute to creating and maintaining inequalities in health and how to intervene to prevent this. Each MoH and DHB working group developed an action plan outlining ways to strengthen their capacity to tackle inequalities in health. Participants considered: how to institutionalize ideas about inequalities in health into their institutions; how their institution is structured to create and maintain health inequalities; how the processes and ways of working used in their institution, and how the rules, legal requirements and formal policies governing their institution create and maintain health inequalities; and how to intervene to change them.

**Figure 1:**
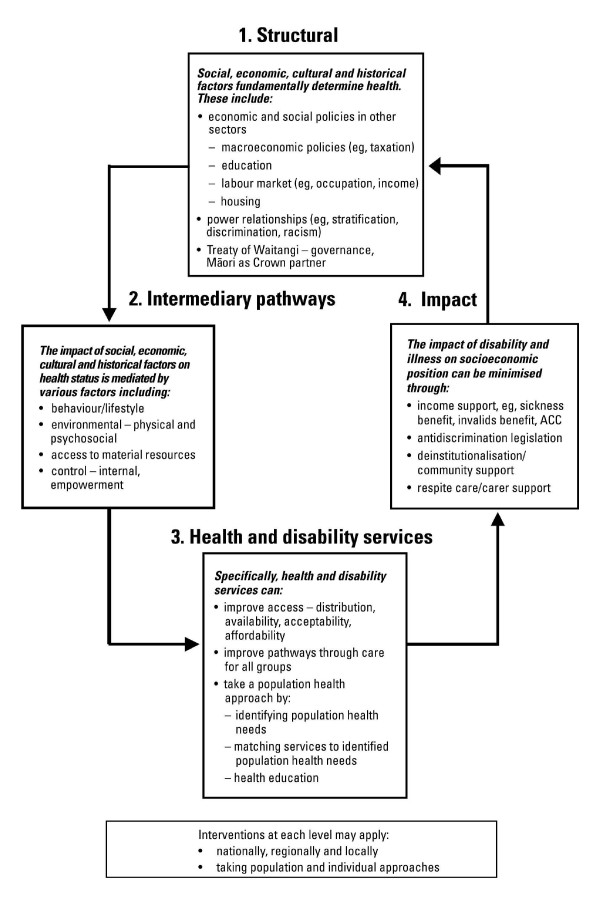
Intervention Framework to Improve Health and Reduce Inequalities.

As part of the project, the team developed a tool, the *Health Equity Assessment Tool *(HEAT),[20] to assist in assessing how particular inequalities in health have developed and where the effective intervention points are to tackle them. It is adapted from part of a health impact assessment tool developed in Wales[21]. This 12-question tool enables rapid assessment of health policy, programmes or services for their current or future impact on health inequalities. The HEAT tool questions are presented in Appendix 1. During the workshops the questions in the tool were applied to a range of health issues, demonstrating the use of the tool in multiple contexts. The HEAT tool includes an *Intervention Framework to Improve Health and Reduce Inequalities *outlined in Figure 1[7]. This *Intervention Framework *describes a comprehensive approach at four levels: structural, intermediary pathways, health and disability services, and impact. Approximately 160 people participated in the workshops, including members of the senior management team of the MoH and most of the 21 DHBs. Some senior staff found it difficult to make themselves available for two-day workshops. As a result other staff members were able to attend.

## Results

Participants were able to identify a range of institutional mechanisms within their health institutions that create or maintain health inequalities and a range of ways to address them. While the emphasis of their discussion was addressing institutionalized racism, their planning ranged more widely. In terms of ideas, they recognised the need to highlight both the problem of health inequalities and how to effectively intervene. In terms of how to institutionalize ideas about inequalities in health into their institutions participants identified: further training of staff, politicians and providers; building inequalities objectives into key strategic and policy documents; and using Maori models of health in policy-making in order to better meet the needs of Maori.

At the structural level participants identified: increased Maori participation and partnership in decision making through shared leadership in policy-making and increased Maori representation in DHBs at the political, executive and workforce levels; strengthening DHB relationships with iwi (tribes) e.g. through memoranda of understanding; and funding and supporting services provided 'by Māori for Māori'.

In terms of institutional processes participants identified: incorporating a strong focus on health inequalities in DHB needs assessment templates; ensuring all DHB patients receive their full benefit entitlement; ensuring a strategic and systematic approach to intersectoral work; and encouraging integral and ongoing community involvement in the work of the institution. Participants also recognised the importance of formal requirements to address inequalities such as mandating the use of the HEAT tool in planning; requiring all evaluations to assess the impact on inequalities (especially on Māori health), and not just on improving overall health; and requiring DHBs to report on their progress in addressing health inequalities as part of the monitoring of their contracts.

The workshop process enabled participants to identify a range of obstacles to tackling inequalities. These included: a perceived lack of leadership across the sector, strong vested interests for the status quo and lack of knowledge about effective interventions. Participants also identified supports required to assist this work. These included: good information, strong relationships between the health sector and other sectors of society, and appropriate accountability and monitoring mechanisms.

**Figure 2:**
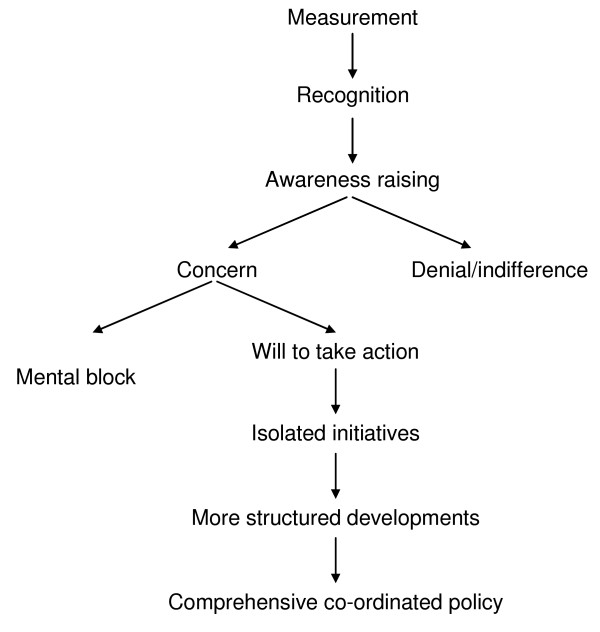
Action Spectrum on Inequalities in Health.

In anonymous written evaluations of the training, 90% of participants rated the workshop as valuable or better. The level of participants' commitment to tackling inequalities was measured at the beginning and end of the workshops using Whitehead's action spectrum on inequalities in health (see Figure 2)[22]. Whitehead argues that countries can move along this spectrum, from measuring health inequalities to recognition of disparities and an awareness of health determinants and consequences. Once awareness is raised, they may be concerned about, deny or be indifferent to inequalities. If there is concern, countries may develop a will to take action and move through a process from isolated initiatives to more structured developments and ultimately to a comprehensive co-ordinated policy. In the workshop evaluations the training team applied Whitehead's model to individuals and found it a useful extension of her work. Nearly all participants moved at least one step and all but two rated themselves as willing to take action following the workshops.

Teleconference follow-up sessions held some months after the workshops provided support to participants to continue to tackle health inequalities. While only 24 participants took part, some people spoke for a wider group from their workplace. Participants in these sessions reported using the HEAT tool and the *Intervention Framework*[7] to better analyse the impact of current, or future, interventions on health inequalities. The *Intervention Framework *was particularly helpful in focusing the health sector on its own role in contributing to, and maintaining, health inequalities. Participants reported they had been able to spread the messages of the workshop to colleagues and had made progress building an inequalities approach within their institutions as a result of the workshop action plans. This included: commitment to accurate ethnicity recording, developing equity-focused planning templates, strengthening relationships with Māori, and the inclusion of training on inequalities in health in MoH policy analyst training courses.

## Discussion

The Government's commitment to tackling inequalities in health was critical to enabling this training programme to proceed and has provided a platform for work to continue. The initiative was greatly strengthened by the linking of academic, policy, and sector experience in the partnership between of the UOW and the MoH. The advantages were the academics' familiarity with the literature and overseas developments, the MoH staff's policy and sector knowledge, and the signal that leadership by the MoH gave to the sector that tackling inequalities in health is a critical issue. Basing the programme on leading national and international research increased the programme's credibility.

The use of locally adapted equity tools as part of the workshops increased participants' familiarity with the tools and may have contributed to the participants' willingness to use them subsequently. Requiring participants to develop action plans at the workshops meant they had a strategy upon which to act when they returned to their workplaces. Using a case study, in this case ethnic inequalities, enabled the presenters to focus the information provided, while teaching participants key lessons that they could apply to other arenas, such as socioeconomic, gender and geographic inequalities. Institutional theory was helpful in providing insights into the ways that drivers of inequalities, such as racism, are built into health institutions and it provided a way to think about effective institutional interventions.

Two-day workshops are a significant time commitment, particularly for senior staff, although they provide the opportunity to thoroughly explore the issue. A one-day workshop has subsequently been developed and delivered. Key aspects of the programme have also been presented in short sessions at existing forums, such as national meetings of DHB managers. Low attendance at the follow-up sessions may be the result of the difficulty of making time to step outside the day-to-day pressures in the sector to take a more strategic approach. We were left with a concern that we were 'preaching to the converted' given that attendance was voluntary. However, we were also conscious of developing a group of health inequalities 'champions' who were in senior positions with considerable influence.

Key recommendations made to the MoH by the training team at the conclusion of the training included: the critical need for continued sector leadership on the importance of tackling inequalities in health; training for all those who work in the health sector; equity-focused contracting and monitoring frameworks; and the development and dissemination of case studies of efforts to tackle inequalities in health. The MoH now contractually requires that DHBs report on progress in tackling inequalities in health using the HEAT tool and the *Intervention Framework*, and early reporting is favourable. A subsequent audit of the use of the tools indicated they play a valuable part in efforts to tackle inequalities[23]. Case studies are currently underway and further training has since been undertaken, including training with Māori health providers.

This series of awareness-raising workshops has made a valuable contribution to the process of moving the New Zealand health sector from isolated initiatives towards the development of comprehensive co-ordinated policy to tackle inequalities in health[22]. Ongoing challenges remain such as providing appropriate levels of support and accountability so that the enthusiasm of those who attended is maintained. There is a real danger that the expectations and hopes raised in this training programme will not be met without clear ongoing government commitment, bipartisan political support, and sector leadership. Institutional, professional, and economic levers have to be utilised to encourage progress and innovation in tackling inequalities.

The task ahead is to ensure an equity approach is institutionalized throughout the health sector. A future challenge is to roll out the programme beyond the health sector to leaders and managers in other sectors with an impact on health such as education, social welfare, and housing. This New Zealand experience provides a model that may be applicable in other jurisdictions.

## Competing interests

The author(s) declare that they have no competing interests.

## Appendix 1

Health Equity Assessment Tool Questions

1. What health issue is the policy/programme trying to address?

2. What inequalities exist in this health area?

3. Who is most advantaged and how?

4. How did the inequality occur? (What are the mechanisms by which this inequality was created, is maintained or increased?)

5. What are the determinants of this inequality?

6. How will you address the Treaty of Waitangi in the context of the New Zealand Public Health and Disability Act 2000?

7. Where/how will you intervene to tackle this issue? Use the Ministry of Health Intervention Framework to guide your thinking.

8. How could this intervention affect health inequalities?

9. Who will benefit most?

10. What might the unintended consequences be?

11. What will you do to make sure it does reduce/eliminate inequalities?

12. How will you know if inequalities have been reduced/eliminated?
